# Mitochondrial calcium uniporter activates TFEB-driven autophagy to promote migration of breast cancer cells

**DOI:** 10.22038/IJBMS.2023.71522.15548

**Published:** 2023

**Authors:** Lin Yuan, Qimou Lin, Fei Shen, Yong Li, Junda Li, Bo Xu

**Affiliations:** 1 Department of General Surgery, The First Affiliated Hospital, Jinan University, Guangzhou, CN 510630, China; 2 Department of Breast, Jiangmen Central Hospital, Jiangmen, CN 529030, China; 3 Departments of General Surgery and Thyroid Surgery, Guangzhou First People’s Hospital, School of Medicine, South China University of Technology, Guangzhou, CN 510180, China

**Keywords:** Autophagy, Breast neoplasms, Migration, Mitochondrial calcium- uniporter, Transcription factor EB

## Abstract

**Objective(s):**

Tumor metastasis is the leading cause of death in breast cancer (BC) patients and is a complicated process. Mitochondrial calcium uniporter (MCU), a selective channel responsible for mitochondrial Ca^2+^ uptake, has been reported to be associated with tumorigenesis and metastasis. The molecular mechanisms of MCU contributing to the migration of BC cells are partially understood. This study investigated the role of MCU in BC cell metastasis and explored the underlying mechanism of MCU-mediated autophagy in BC cell migration.

**Materials and Methods:**

The Kaplan-Meier plotter database was used to analyze the prognostic value of MCU mRNA expression. Western blotting was used to examine the expression level of MCU in 4 paired BC and adjacent normal tissues. The cellular migration capability of BC was measured by transwell migration assay and wound healing assay. Western blotting and reverse transcription-quantitative polymerase chain reaction were performed to detect the expression levels of autophagy-related markers. The effects of MCU activation or inhibition on TFEB nuclear translocation in BC cells were detected by laser scanning confocal microscopy.

**Results:**

Expression of MCU was found to be negatively correlated with BC patient prognosis in the Kaplan-Meier plotter database. Compared with the adjacent normal tissues, MCU was markedly up-regulated in the BC tissues. MCU overexpression promoted cellular migration, activated autophagy, and increased TFEB nuclear translocation in BC cells, whereas its knockdown produced the opposite effects.

**Conclusion:**

MCU activates TFEB-driven autophagy to promote BC cell metastasis and provides a potential novel therapeutic target for BC clinical intervention.

## Introduction

Breast cancer (BC) is one of the most common malignancies among women globally and is the second leading cause of cancer-related deaths (1). It has been reported that 20–30% of BC patients may develop metastases after primary diagnosis and treatment, and approximately 90% of cancer-related deaths are attributed to metastasis (2). Although there has been progress in the diagnosis and treatment of BC, the mechanism underlying BC metastasis remains unclear. Thus, it is essential to explore the molecular mechanisms that contribute to the migration of BC cells.

Many studies have demonstrated that the tumor microenvironment plays a fundamental role in tumor cell metastasis (3, 4). The tumor microenvironment can stimulate the genetically regulated programs to help tumor cells overcome metabolic stress, maintain homeostasis, and survive in the poor physical environment (5). Autophagy is a conserved, self-degradation system that was originally recognized as an important pro-survival mechanism that supplies cells with nutrients under unfavorable growth conditions (6). In a nutrient-deprived growth environment, tumor cells are more reliant on autophagy for survival than normal cells due to their rapid growth rates (7). It has been suggested that autophagy can promote tumor cell survival by conferring resistance to anticancer drugs and favoring the survival of dormant cancer cells (8). Autophagic processes are controlled by several regulators, such as mammalian target of rapamycin (mTOR) and transcription factor EB (TFEB). TFEB is a central regulator of lysosomal biogenesis and autophagy that functions by regulating coordinated lysosomal expression and gene network regulation (9). 

Mitochondrial calcium uniporter (MCU), a highly selective Ca^2+^ channel protein of the inner membrane of mitochondria (10), contains a proteolytically cleaved mitochondrial import sequence, two coiled-coil domains, two transmembrane domains, and a short motif of amino acids between the two transmembrane domains critical for Ca^2+^ transport (11). Mitochondria regulate Ca^2+^-dependent cell functions, including gene transcription, autophagy, cell motility, and survival through Ca^2+^ uptake/extrusion (12). It has been widely accepted that the MCU level in tumor cells positively correlates with mitochondrial Ca^2+^ uptake, ROS production, migratory capacity, and propensity for metastatic dissemination (13). Mitochondrial Ca^2+^ uptake regulates autophagy; however, whether it plays a permissive or inhibitory role depends on the cell context (14-16). Although the biological role of MCU in the progression of several cancer types has been studied, it remains unclear whether MCU is involved in BC cell metastasis via the regulation of autophagy. 

In this study, we investigated the role of MCU in BC cell metastasis. In addition, the underlying mechanism of MCU**-**mediated autophagy in BC cell metastasis was intensely explored. Our study reveals a novel underlying mechanism of tumor metastasis and provides a potential therapeutic strategy for BC patients.

## Materials and Methods 

***Clinical tissues and cell culture***

Tumors and adjacent normal tissues were collected from BC patients who underwent resection in our hospital. The human breast adenocarcinoma cell lines MDA-MB-231 (CVCL_0062), BT-549 (CVCL_1092), BT-474 (CVCL_0179), MCF-7 (CVCL_0031), and T-47D (CVCL_0553) were purchased from the American Type Culture Collection (ATCC, Manassas, USA). SUM-159-PT (CVCL_5423) was purchased from Procell Life Science Technology (Wuhan, China). MDA-MB-231 and MCF-7 cells were maintained in Dulbecco’s modified Eagle’s medium (DMEM; Gibco, Grand Island, USA) supplemented with 10% fetal bovine serum (FBS; Thermo Fisher Scientific, Waltham, USA) while the others were maintained in RPMI-1640 medium (Gibco, Grand Island, USA) supplemented with 10% FBS. The cells were cultured at 37 ^°^C in a humidified atmosphere containing 5% CO_2_ (17).

***Transfection and chemical treatment***

pDEST47-MCU-GFP (plasmid #31732) was supplied by Addgene (Cambridge, USA). siRNA was synthesized by GenePharma Company (Shanghai, China). The sense and antisense strands of MCU-siRNA were CCUAGAGAAAUACAAUCAACUCAdAdG and CUUGAGUUGAUUGUAUUUCUCUAGGUC. pcDNA3.1 plasmid and a scrambled siRNA were used as negative controls. MDA-MB-231 cells were seeded in 6-well plates until they reached 80% confluence for transfection of plasmids or siRNAs with Lipofectamine® 3000 reagent (Thermo Fisher Scientific, Carlsbad, USA) according to the manufacturer’s protocols. Chloroquine (CQ; 10 μM; Sigma, St Louis, USA) was diluted in H_2_O. Ruthenium red (Rured; 10 μM; Sigma, St Louis, USA) and spermine (Sper; 10 μM; Sigma, St Louis, USA) were dissolved in dimethyl sulfoxide (DMSO; Sigma, St Louis, USA). MDA-MB-231 cells were treated with Rured or Sper for 48 hr. 

***Reverse transcription-quantitative polymerase chain reaction (qRT*** ***‐*** ***PCR)***

Based on Rio *et al*., TRIzol (Invitrogen, Carlsbad, CA, USA) was used to isolate the total cellular RNA (18). RNA extraction was carried out according to the manufacturer’s instructions. To detect the mRNA levels of related genes, cDNA was reverse transcribed by a RevertAid First Strand cDNA Synthesis kit (Thermo Fisher Scientific, Carlsbad, USA). Next, qRT-PCR was performed using the FastStart Universal SYBR Green Master kit (Roche, Mannheim, Germany) with an ABI7500 PCR instrument (Applied Biosystems, Foster City, USA). The thermocycling steps were as follows: 5 min at 95 ^°^C, followed by 40 cycles at 95 ^°^C for 15 sec and 60 ^°^C for 30 sec. ΔΔCT values were determined from the mean CT values of three technical replicates per sample in each group. To determine the expression level, qRT‒PCR was performed using the following forward and reverse primers: LC3-II (F: 5’-GCTTGCAGCTCAATGCTAAC-3’, R: 5’-CCTGCGAGGCATAAACCATGTA-3’); 

LAMP1 (F: 5’-CCTACGAGACTGCGAATGGT-3’, 

R: 5’-CCACAAGAACTGCCATTTTTCT-3’);GAPDH (F: 5’-GGAGTCAACGGATTTGGTCGTATTG-3’, 

R: 5’-TCTCGCTCCTGGAAGATGGTGAT-3’); 

***Western blot (WB) analysis***

Tissues or cells were lysed with cell lysis buffer (Cell Signaling Technology, Beverly, USA) according to the manufacturer’s instructions. The lysates were centrifuged at 14000** × **g for 15 min at 4 ^°^C. The protein concentration of the supernatant was determined using a bicinchoninic acid assay kit (Kangwei Technology, Beijing, China). Equal amounts of protein (30 µg/lane) were separated in 10% SDS–PAGE gels and transferred to polyvinylidene fluoride (PVDF; Merck, IPFL00010, Schwal-bach, Germany) membranes, which were blocked with 5% nonfat milk in TBS with 0.1% Tween 20 for 2 hr at room temperature. Membranes were incubated with primary antibodies against GAPDH (1:1000; Cell Signaling Technology, cat.5174, Beverly, USA), Lamin B1 (1:1000; Cell Signaling Technology, cat.13435, Danvers, USA), β-Actin (1:1000; Cell Signaling Technology, cat.4970, Danvers, USA), LC3A/B (1:1000; Cell Signaling Technology, cat.12741, Danvers, USA), LAMP1 (1:1000; Cell Signaling Technology, cat.3243, Danvers, USA), TFEB (1:1000; Cell Signaling Technology, cat.37785, Danvers, USA), MCU (1:1000; Cell Signaling Technology, cat.14997, Danvers, USA), and p62 (1:1000; Cell Signaling Technology, cat.39749, Danvers, USA) at 4 ^°^C overnight. After three washes with TBS-Tween 0.1% buffer, we incubated the membranes with anti-rabbit IgG, HRP-linked antibody (1:2000; Cell Signaling Technology, cat.7074, Beverly, USA) and anti-biotin, HRP-linked antibody (1:2000; Cell Signaling Technology, cat.7075, Beverly, USA) for 1 hr at room temperature. The membranes were then visualized using the enhanced chemiluminescence system (19, 20).

***Wound healing assay***

After 48 hr of treatment with Sper, Rured or DMSO, MDA-MB-231 cells were seeded in a 6-well plate (5×10^5 ^cells/well). Cell proliferation can be inhibited by thymidine (21). In the proliferation inhibition groups, cells were treated with thymidine (10 μM, Sigma, St. Louis, USA) for 24 hr after seeding. To perform the experiment, a 100 μl sterile pipette tip was used to lightly line. After rinsing the cells with phosphate-buffered saline (PBS; Thermo Fisher Scientific, Waltham, USA) 3 times, the cells were allowed to heal the wounds for 24 hr and 48 hr in an FBS-free medium. In the proliferation inhibition groups, 10 μM thymidine was added to the medium. After 24 hr and 48 hr, the wound areas were photographed and calculated by measuring the average width of the wound (22).

***Transwell migration assay***

Transwell chambers (24 wells; pore size 8 μm; Corning, NY, USA) were used to detect cell migration. MDA-MB-231 cells (1×10^5^) were seeded in the upper chambers in 100 μl of serum-free medium. The lower chambers were filled with 500 μl of medium containing 10% FBS. The chamber was incubated at 37 ^°^C for 24 hr. In the proliferation inhibition groups, 10 μM thymidine was added to the medium. Cells on the inside of the transwell inserts were removed with a cotton swab, and cells on the underside of the insert were fixed with 4% paraformaldehyde, stained with crystal violet, and then visualized and counted. Cell migration ability was determined by calculating the number of cells passing through the membrane in five microscopic fields per well, and the extent of migration was expressed as the average number of cells per microscopic field (23).

***Laser scanning confocal microscopy (LSCM)***

For the immunofluorescence study, MDA-MB-231 cells were grown on glass coverslips for 1 day. After washing with PBS, the cells were fixed using frozen methanol (-20 ^°^C) for 10 min, washed 3 times in PBS, permeabilized with 0.5% Triton X-100 (Sigma, St. Louis, USA) in PBS for 10 min, washed 3 times in PBS and blocked in 3% BSA for 30 min. The slides were incubated with primary antibodies against TFEB (1:100; Thermo Fisher Scientific, cat.PA5-96632, Waltham, USA) and LC3B (1:100; Thermo Fisher Scientific, cat.PA5-115501, Waltham, USA) in 3% BSA overnight at 4 ^°^C and washed 3 times in PBS. Cells were then incubated with goat anti-rabbit Alexa Fluor Plus 488 antibody (1:1000; Thermo Fisher Scientific, cat.A32731, Waltham, USA) in 3% BSA for 60 min at room temperature. To mark the nucleus, the cells were again stained with DAPI (1:500; Sigma, Zwijndrecht, Netherlands). An Olympus FV1000 confocal microscope with a 60X objective was used to capture images (24, 25).

***Statistical analysis***

The data are presented as the mean±standard deviation (SD) of five independent experiments. A double-sided Student’s t-test was used to analyze the differences between the samples. Matched samples were tested with a paired, double-sided t-test and rank correlation. The statistical analysis was performed using SPSS software (version 25.0). A *P*-value<0.05 was considered statistically significant.

## Results

***Overexpression of MCU predicts the poor prognosis of BC***

**Figure 1:**
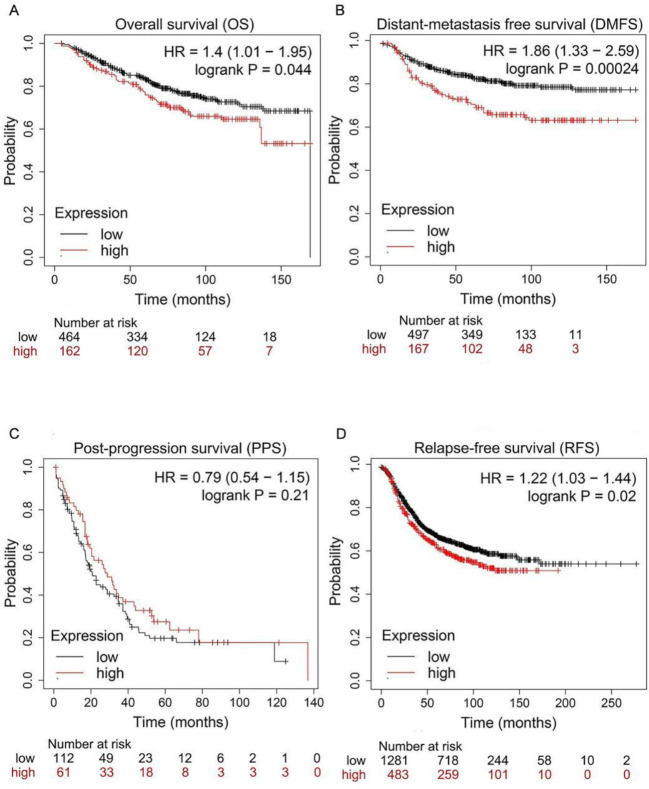
Mitochondrial calcium uniporter (MCU) expression correlates with the prognosis of BC patients

To assess the role of MCU in the survival and prognosis of BC patients, we used Kaplan‒Meier plotter (http://kmplot.com/analysis/) to analyze the prognostic values of the mRNA expression of MCU. For OS and PPS, patients who were alive at the last follow-up or lost to follow-up were defined as censored data. For other survival analyses, patients who had not experienced the event (distant metastasis for DMFS and tumor recurrence for PFS) or died of other causes or if they were lost to follow-up were defined as censored data. As was shown in Figure 1, the expression of MCU was significantly associated with BC patient prognosis. The results showed that BC patients with higher MCU expression exhibited poorer overall survival (OS) (HR=1.40, 95% CI: 1.01-1.95, *P*=0.044; Figure 1A), distant metastasis-free survival (DMFS) (HR=1.86, 95% CI: 1.33-2.59, *P*=0.00024; Figure 1B), and relapse-free survival (RFS) (HR=1.22, 95% CI: 1.03-1.44, *P*=0.02; Figure 1D).

***Expression of MCU is positively associated with cellular invasiveness of BC***

**Figure 2:**
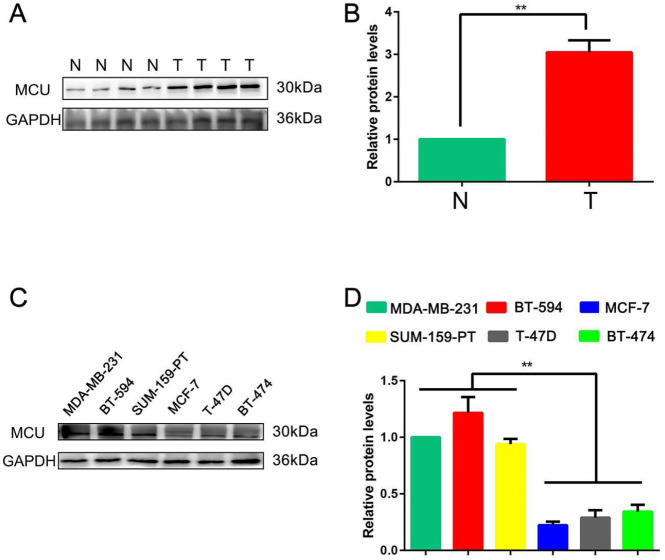
Mitochondrial calcium uniporter (MCU) expression is positively associated with cellular invasiveness of breast cancer (BC)

After the expression of MCU was found to be negatively correlated with BC patient prognosis from the Kaplan‒Meier plotter database, we performed our research with clinical samples to determine the biological role of MCU in the tumor progression of BC. WB analysis was used to examine the expression level of MCU in 4 paired BC and adjacent normal tissues. The results revealed that MCU was markedly up-regulated in BC tissues at the protein level compared with adjacent normal tissues (*P*<0.01; Figure 2A, B).

To validate the results of the Kaplan‒Meier plotter database, MCU expression was measured by WB analysis in 6 BC cell lines (MDA-MB-231, BT-549, SUM-159-PT, BT-474, MCF-7, and T-47D). Our data showed that the expression level of MCU in BC cell lines with higher invasive potential, such as MDA-MB-231, BT-549, and SUM-159-PT, was significantly higher than that in MCF-7, T-47D and BT-474 cells (*P*<0.01; Figure 2C, D). This outcome revealed a consistently differential expression with the Kaplan‒Meier plotter database, suggesting that MCU expression is higher in the BC subtypes with poorer prognosis.

All these results indicated that MCU may mediate and promote the occurrence and development of BC.

In our analysis above, MDA-MB-231 exhibited high expression level of MCU in the 6 BC cell lines. Considering that MDA-MB-231 is widely used in BC research, we chose it to investigate the underlying mechanisms of MCU in mediating BC cell migration capacity in further studies. 

***Activation of MCU enhances the migratory capability of BC cells***

**Figure 3:**
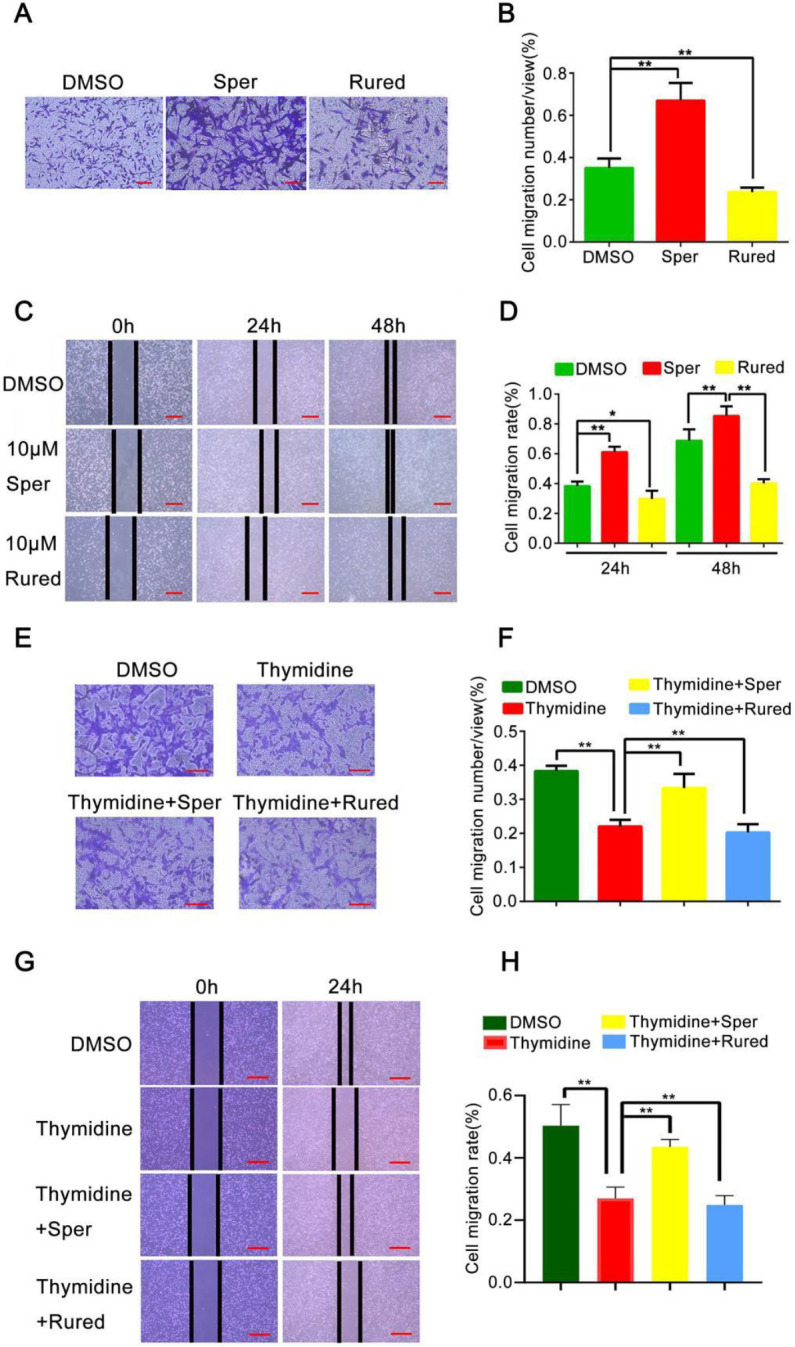
Activation of mitochondrial calcium uniporter (MCU) promotes migration ability of breast cancer (BC) cells

As the expression of MCU was found to be associated with the invasive potential of BC cells, we then explored whether MCU exerted an effect on mediating the cellular migration capability of BC. Under physiological conditions, MCU can be activated by Sper and inhibited by Rured (26). Transwell migration assays were performed to investigate the migration capacity of BC cells. As shown in the results, the migration of MDA-MB-231 cells treated with Sper increased significantly compared with the control group whereas the migration of the Rured group decreased noticeably in comparison with the control group (both *P*<0.01, Figure 3A, B). Furthermore, a wound-healing assay was also carried out. We found that the wound distance was markedly decreased after 48 hr in BC cells treated with Sper. Conversely, repressed MCU significantly increased the wound distance in BC cells treated with Rured (Figure 3C, D). To suppress the effect of cell proliferation on the experimental results, we used thymidine as a cell proliferation inhibitor. The results of transwell migration and wound healing assays showed that Sper reversed the cell migration inhibition caused by thymidine, and Rured aggravated this effect of thymidine (all *P*<0.01, Figure 3, E-H).

***Overexpression of MCU enhances the migratory capability of BC cells***

**Figure 4:**
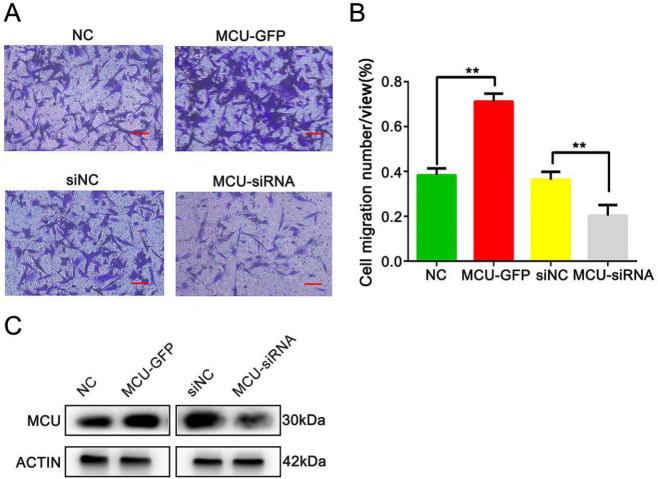
Overexpression of mitochondrial calcium uniporter (MCU) promotes the migration ability of breast cancer (BC) cells

To further verify this result, we constructed MDA-MB-231 stable expression cell lines with MCU silencing or overexpression. MDA-MB-231 cells were transfected with pDEST47-MCU-GFP or MCU-siRNA to selectively increase or knock down MCU protein expression. The transwell migration assay results showed that the migratory capability of MDA-MB-231 cells was significantly promoted after transfection with pDEST47-MCU-GFP. However, it was significantly inhibited after infection with MCU-siRNA (both *P*<0.01, Figure 4A, B). 

***MCU activates autophagy in BC cells***

**Figure 5:**
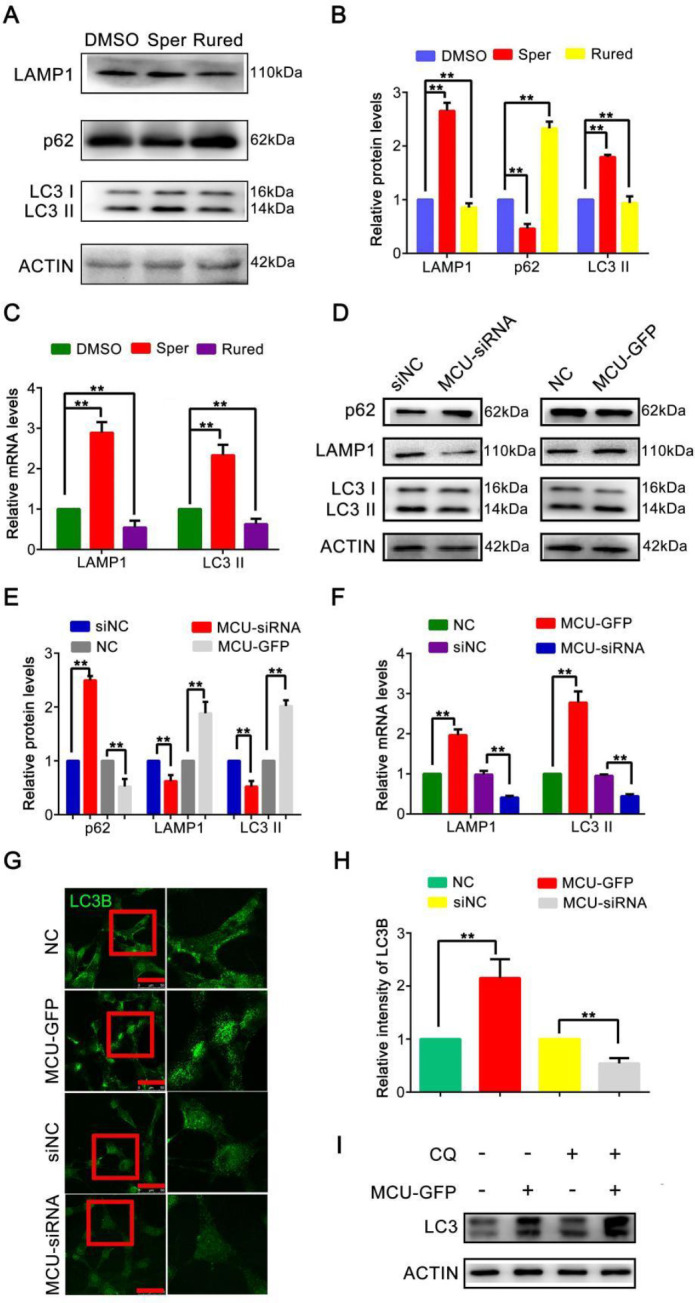
Mitochondrial calcium uniporter (MCU) activates autophagy in breast cancer (BC) cells

As shown in previous studies, autophagy plays a critical role in promoting tumor metastasis (27). To confirm whether MCU promoted the migration of BC cells by inducing autophagy, we performed WB analysis to examine the expression levels of autophagy-related markers (LC3-II, LAMP1, and p62) in MDA-MB-231 cells up- and down-regulated MCU expression. We found that Sper significantly activated the expressions of LC3-II and LAMP1, and inhibited the expression of p62 simultaneously. Opposing changes were observed after MDA-MB-231 cells were treated with Rured (all *P*<0.01, Figure 5A, B). In addition, we verified this result with qRT‒PCR analysis. The data revealed that the mRNA expression levels of LC3-II and LAMP1 were noticeably up-regulated in MDA-MB-231 cells treated with Sper (both *P*<0.01, Figure 5C). Similar results were found in MCU-overexpressing MDA-MB-231 cells (all *P*<0.01, Figure 5D-F). LC3B plays a crucial role in functional autophagosome formation (28). To observe the changes **in** autophagy, we used LSCM to detect the changes in LC3B. In MCU-overexpressing cells, the expression of LC3B was increased significantly (*P*<0.01, Figure 5G, H). LC3-II can accumulate by increasing autophagosome formation or blocking the autophagosome-lysosome fusion process. To distinguish between these two possibilities, CQ was used to block autophagosome-lysosome fusion (29). As Figure 5I shows, after treatment with CQ (10 μM, 4 hr), the level of LC3-II was further enhanced. All these findings suggest that up-regulation of MCU increases the level of autophagy in BC cells.

***MCU activates TFEB-driven autophagy to promote the migration of BC cells***

**Figure 6:**
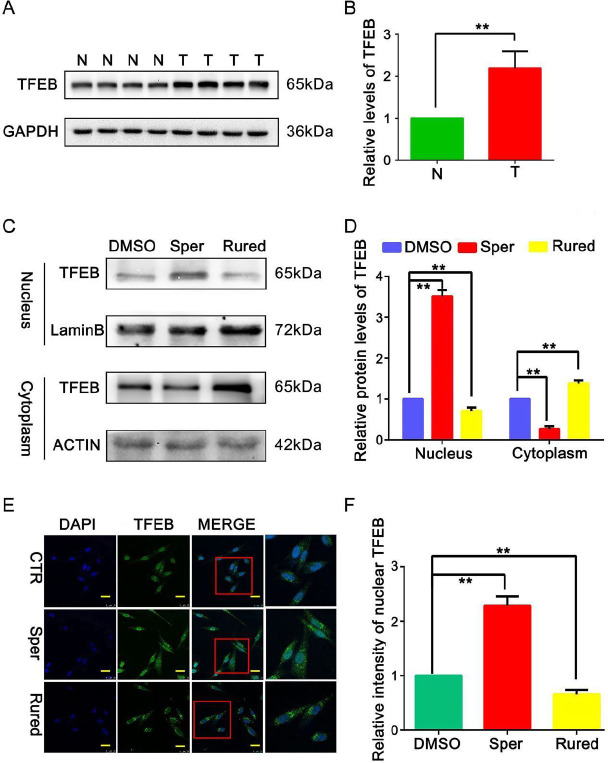
Mitochondrial calcium uniporter (MCU) activates transcription factor EB (TFEB)-driven autophagy to promote migration of breast cancer (BC) cells

As TFEB is a key regulator of autophagy, we used **a** WB assay to examine the expression levels of TFEB in BC and adjacent normal tissues. Compared with the adjacent normal tissues, the expression of TFEB was significantly increased in the BC tissues (*P*<0.05; Figure 6A, B), suggesting that TFEB expression was associated with the tumor progression of BC.

To investigate the functional role of MCU in mediating TFEB-driven autophagy, the expression of TFEB in MDA-MB-231 cells after activating and inhibiting MCU was measured by WB analysis. As shown in Figure 6C and D, activation of MCU could effectively improve the expression of TFEB in the cell nucleus, while the opposite finding was observed after MCU was inhibited. The changes indicated that up-regulation of MCU might increase TFEB nuclear translocation in BC cells. Furthermore, we used LSCM to detect the nuclear translocation of TFEB. As expected, in MDA-MB-231 cells, the nuclear translocation of TFEB was significantly increased after activating MCU with Sper, and it decreased after MCU was inhibited by Rured (Figure 6E, F). Taken together, these data reveal that MCU activates TFEB-driven autophagy to promote BC cell migration. 

The effect of MCU expression on the survival data of breast cancer (BC) patients was examined using the Kaplan-Meier plotter for BC. (A) Overexpression of MCU was associated with lower overall survival (OS) of BC patients. (B) Overexpression of MCU was associated with lower distant metastasis-free survival (DMFS) in BC patients. (C) Expression of MCU was not correlated with post-progression survival (PPS). (D) Overexpression of MCU was associated with lower relapse-free survival (RFS) in BC patients

(A, B) Western blot results showed that MCU was markedly up-regulated in the BC tissues compared with the adjacent normal tissues. Values are expressed as mean±SD, n=4, **P<0.01. (C, D) Expression levels of MCU in 6 BC cell lines (MDA-MB-231, BT-549, SUM-159-PT, BT-474, MCF-7, and T-47D) measured by WB. Values are expressed as mean±SD, n=5, *P<0.05; **P<0.01; ***P<0.001; ****P<0.0001

(A, B) Transwell migration assay results showed the effects of MCU activation or inhibition on BC cell invasion. Scale bar (red line): 50 μm. (C, D) Wound healing assay result showing the effects of MCU activation or inhibition on BC cell migration. Scale bar (red line): 250 μm. (E, F) Transwell migration assay result showed the effects of MCU activation or inhibition combined with 10 μM thymidine on BC cell invasion. Scale bar (red line): 50 μm. (G, H) Wound healing assay result showing the effects of MCU activation or inhibition combined with 10 μM thymidine on BC cell migration. Scale bar (red line): 250 μm. Values are expressed as mean±SD, n=5, *P<0.05; **P<0.01

(A, B) Transwell migration assay showed the effects of MCU overexpression or knockdown on BC cell invasion. Scale bar (red line): 50 μm. (C) Western blot showing the expression levels of MCU and ACTIN in MDA-MB-231 cells transfected with pDEST47-MCU-GFP or MCU-siRNA. Values are expressed as mean±SD, n=5, **P<0.01

(A, B) Western blot showing the expression levels of LAMP1, LC3-II, and p62 in MDA-MB-231 cells treated with Sper or Rured. (C) qRT-PCR analysis result showing the expression levels of LAMP1 and LC3-II in MDA-MB-231 cells treated with Sper or Rured. (D, E) Western blot showing the expression levels of LAMP1, LC3-II, and p62 in MDA-MB-231 cells transfected with pDEST47-MCU-GFP or MCU-siRNA. (F) qRT-PCR analysis result showing the expression levels of LAMP1 and LC3-II in MDA-MB-231 cells transfected with pDEST47-MCU-GFP or MCU-siRNA. (G, H) Effects of MCU silence or overexpression on LC3B in BC cells were detected by LSCM. MDA-MB-231 cells were incubated with LC3B antibody and then labeled with Alexa Fluor Plus 488 antibodies (green). Scale bar (red line): 50 μm. (I) LC3-II expression was determined by western blot analysis in MDA-MB-231 cells treated with pDEST47-MCU-GFP or CQ as indicated. Values are expressed as mean±SD, n=5, **P<0.01

(A, B) Western blot results showed that TFEB was markedly improved in the BC tissues compared with the adjacent normal tissues. (C, D) Western blot results showing the effects of MCU activation or inhibition on TFEB expression in the nuclei of BC cells. (E, F) Effects of MCU activation or inhibition on TFEB nuclear translocation in BC cells were detected by laser scanning confocal microscopy (LSCM). MDA-MB-231 cells were incubated with TFEB Antibody and then labeled with Alexa Fluor Plus 488 antibodies (green). Nuclei were stained with DAPI (blue). The images were captured at 60X magnification. Scale bar (yellow line): 25 μm. Values are expressed as mean±SD, n=5, **P<0.01

## Discussion

As a vital Ca^2+^ channel located in the inner mitochondrial membrane, MCU has been reported to be associated with tumorigenesis (30). In this study, we explored the actions of MCU on tumor progression in BC cells and obtained two major results. First, we have demonstrated that up-regulation of MCU promotes BC cell migration. More importantly, we have established a link between the up-regulation of MCU and BC cell migration by activating TFEB-driven autophagy, which provides a novel mechanism to understand MCU-mediated BC cell metastasis.

Recent studies have found that MCU is highly expressed in colon cancer (31), hepatocellular carcinoma (32), and pancreatic ductal adenocarcinoma (33). Data from the Kaplan‒Meier plotter database revealed that MCU overexpression predicts poor OS, RFS, and DMFS in patients with BC, supporting that MCU promotes the progression of BC. We found that MCU was significantly up-regulated in BC tissues compared with adjacent normal tissues. Similar results have been obtained in other types of cancer (34), meaning that deregulation of MCU plays an important role in carcinogenesis including BC. Richard *et al*. performed modified Boyden chamber assays to measure the invasive potential of 30 BC cell lines and found that MDA-MB-231, BT-549, SUM-159-PT, SUM-149-PT, HCC-1500, and HBL-100 were more aggressive than others (35). Previous studies emphasized that MCU was highly expressed in ER-, basal-like, and invasive BC (36, 37). In our data, the expression level of MCU was higher in MDA-MB-231, BT-549, and SUM-159-PT cells, suggesting that MCU might positively regulate the invasiveness of BC cells.

Sun *et al*. reported that MCU promotes colon cancer metastasis (31). As metastasis is a key factor that contributes to tumor progression, it is necessary to determine the role of MCU in mediating the migration ability of BC cells. The results of transwell migration and wound healing assays showed that activation of MCU increased the cellular migration capability of BC. 

Furthermore, we were motivated to investigate the mechanism of MCU in regulating BC cell migration. Previous studies have indicated that MCU regulates tumor growth and invasiveness through multiple mechanisms. For instance, Xiao *et al*. reported that MCU could activate HIF-1α, VEGF, and EMT pathways and promote the migration and invasion of gastric cancer cells (38). Another study suggested that MCU interacts with RIPK1 to promote colorectal cancer cell proliferation by increasing mitochondrial Ca^2+^ uptake and energy metabolism (39). Here, we explored the mechanism by which MCU regulates BC cell migration from the perspective of tumor cell autophagy. Autophagy is an evolutionarily conserved catabolic process that is critical for maintaining cellular homeostasis during stress conditions. Dysregulated autophagy has implications for health and disease. As autophagy has been proven to promote BC metastasis via various mechanisms and identified as a target for therapeutic intervention in BC (40-43), we examined whether MCU could regulate autophagy in BC cells. In our research, we chose three autophagy-related markers (LC3-II, LAMP1, and p62) and measured their expression levels to reflect autophagy changes in MCU-activated or MCU-inhibited BC cells. LC3-II, the lipidated form of LC3, is embedded in the autophagosome membrane, and its levels are proportional to the number of autophagosomes. LAMP1 is a major protein component of the lysosomal membrane and it is thought to be an important regulator of the successful maturation of both autophagosomes and phagosomes (28). P62 is an autophagy receptor and a selective substrate for autophagy, which delivers polyubiquitinated cargoes to autophagy and accumulates in autophagy**-**deficient cells (44). In our data, opposite expression levels of autophagy-related markers were detected in different statuses of MCU expression. Likewise, the mRNA expression levels of LC3-II and LAMP1 were significantly up-regulated after activating MCU with Sper. LC3B is vital for the execution of autophagy and is a widely accepted marker for assessing autophagy activity. We detected the expression changes of LC3B and found that it was increased significantly in MCU-overexpressing cells. These findings indicate that MCU has bidirectional regulatory effects on autophagy in BC cells. Overexpression of MCU activates autophagy to promote tumor cell migration.

It is generally well-accepted that TFEB is the second-most characterized member of the MiT family and is dysregulated in many cancers. Researchers have shown that TFEB is overexpressed in renal cell cancer and is associated with aggressive biological behavior (45). According to Giatromanolaki *et al*., enhanced TFEB expression is related to aggressive clinical features and can be an unfavorable independent prognostic factor in BC (40). Under normal conditions, TFEB is localized to the cytoplasm. Under aberrant lysosomal storage conditions, TFEB translocates from the cytoplasm to the nucleus, resulting in the activation of lysosomal and autophagic genes (46, 47). TFEB-driven autophagy has been proven to potentiate TGF-β induced migration in pancreatic cancer cells (48). In our study, up-regulated MCU increased the expression of TFEB in the cell nucleus, indicating that MCU modulates TFEB localization. Furthermore, by LSCM, an increase in TFEB nuclear translocation was clearly observed after MCU was activated, whereas the opposite change was detected in MCU-inhibited cells, suggesting that MCU could activate TFEB-driven autophagy to promote tumor cell migration.

Although many validation experiments have been performed, our study lacks strong evidence to prove the changes in MCU in BC patients. In addition, there is a lack of nude mouse tumorigenesis experiments to verify the role of potential targets. Together, our work demonstrated that MCU induces autophagy to promote BC cell migration via TFEB. Targeting MCU could be a potential treatment strategy for BC.

## Conclusion

Our research showed the relationship between MCU and BC cell invasion. Overexpression of MCU predicts poor prognosis of BC patients. MCU activates TFEB-driven autophagy to promote migration in BC cells, which provides a new target for BC therapy.

## Authors’ Contributions

L Y and B X contributed to the design and review of the research. F S, QM L, and L Y performed experiments and collected data. Y L, JD L, and B X analyzed the data and discussed the results. B X supervised, directed, and managed the study. L Y wrote the article. L Y and B X revised the article. L Y, QM L, F S, Y L, JD L, and B X read and approved the final version of the article.

## Data Availability

The data used to support the findings of this study are included in the article.

## Conflicts of Interest

The authors declare that they have no conflicts of interest.
